# A biomathematical model of atherosclerosis in mice

**DOI:** 10.1371/journal.pone.0272079

**Published:** 2022-08-03

**Authors:** Sibylle Schirm, Arash Haghikia, Markus Brack, Peter Ahnert, Geraldine Nouailles, Norbert Suttorp, Markus Loeffler, Martin Witzenrath, Markus Scholz

**Affiliations:** 1 Institute for Medical Informatics, Statistics and Epidemiology, University of Leipzig, Leipzig, Germany; 2 Department of Cardiology, Charité – Universitätsmedizin Berlin, Campus Benjamin Franklin, Berlin, Germany; 3 DZHK (German Center for Cardiovascular Research), Partner Site Berlin, Berlin, Germany; 4 Charité – Universitätsmedizin Berlin, Corporate Member of Freie Universität Berlin and Humboldt-Universität zu Berlin, Division of Pulmonary Inflammation, Berlin, Germany; 5 Charité – Universitätsmedizin Berlin, Corporate Member of Freie Universität Berlin and Humboldt-Universität zu Berlin, Department of Infectious Diseases and Respiratory Medicine, Berlin, Germany; 6 LIFE Research Center of Civilization Diseases, University of Leipzig, Leipzig, Germany; 7 Berlin Institute of Health (BIH), Berlin, Germany; Ludwig-Maximilians-Universitat Munchen, GERMANY

## Abstract

Atherosclerosis is one of the leading causes of death worldwide. Biomathematical modelling of the underlying disease and therapy processes might be a useful aid to develop and improve preventive and treatment concepts of atherosclerosis. We here propose a biomathematical model of murine atherosclerosis under different diet and treatment conditions including lipid modulating compound and antibiotics. The model is derived by translating known biological mechanisms into ordinary differential equations and by assuming appropriate response kinetics to the applied interventions. We explicitly describe the dynamics of relevant immune cells and lipid species in atherosclerotic lesions including the degree of blood vessel occlusion due to growing plaques. Unknown model parameters were determined by fitting the predictions of model simulations to time series data derived from mice experiments. Parameter fittings resulted in a good agreement of model and data for all 13 experimental scenarios considered. The model can be used to predict the outcome of alternative treatment schedules of combined antibiotic, immune modulating, and lipid lowering agents under high fat or normal diet. We conclude that we established a comprehensive biomathematical model of atherosclerosis in mice. We aim to validate the model on the basis of further experimental data.

## Introduction

Despite decades of intensive research, atherosclerosis is still one of the most common causes of death worldwide [1] and the asymptomatic prevalence in the population is generally high [2]. Atherosclerosis is a disease starting in the intima, mainly affecting middle-size and larger arteries, e.g. the coronary and the carotid arteries. Disease pathology starts with endothelial dysfunction increasing permeability of the intima. Lipoproteins invading the intima induce an immune response marked by monocytes invasion and transformation into inflammatory macrophages. These macrophages take up the lipids, develop into foam cells and undergo apoptosis, thereby establishing a vicious circle of inflammation with further recruitment of immune cells. This process leads to formation of an atherosclerotic plaque and to a progressive occlusion of arteries. As a consequence, atherosclerosis is characterized by a state of chronic inflammation [3]. At an advanced stage, the plaque can develop into a larger lesion consisting of cholesterol deposits enclosed in a fibrous cap [4]. In the final stage, rupture or erosion of the fibrous cap can induce acute intravascular thrombosis with complete occlusion of the blood vessel and trigger severe complications such as myocardial infarction or stroke [4].

Several factors of atherosclerosis development are known, most prominently age, high blood levels of atherogenic cholesterols, diabetes mellitus, high blood pressure and smoking. Further pro-inflammatory triggers also accelerate this process [3] summarized as inflammatory residual risk. More recently, disturbance of gut microbiome was identified as another risk factor [5]. Thus, immuno-modulating or antibiotic drugs can affect atherosclerosis formation. Prevention of atherosclerosis is typically pursued by eliminating or ameliorating modifiable risk factors, most importantly lipid lowering medication [6].

Due to the complex biological process of atherosclerotic plaque formation including several stimulating and modulating factors, development of optimal atherosclerosis prevention and treatment is a non-trivial task. Biomathematical models can support this process by performing systematic simulations of possible treatment strategies and selecting the most promising ones. Several such models were proposed in the past considering different aspects of the underlying physiological process such as oxidative stressors [7, 8], impact of growth factors and smooth muscle cells [9, 10], necrotic cores [11] or medication effects [12, 13]. We here propose a mechanistic model of atherosclerosis formation in mice and parametrize it on the basis of a rich resource of literature data and our own experimental data comprising dietary interventions, lipid lowering agents, antibiotics and immune-modulating therapy. Our aim is to find mechanistic explanations for the effects of these interventions that allow us to simulate alternative scenarios.

## Methods

### General structure of the model

**Fig 1:**
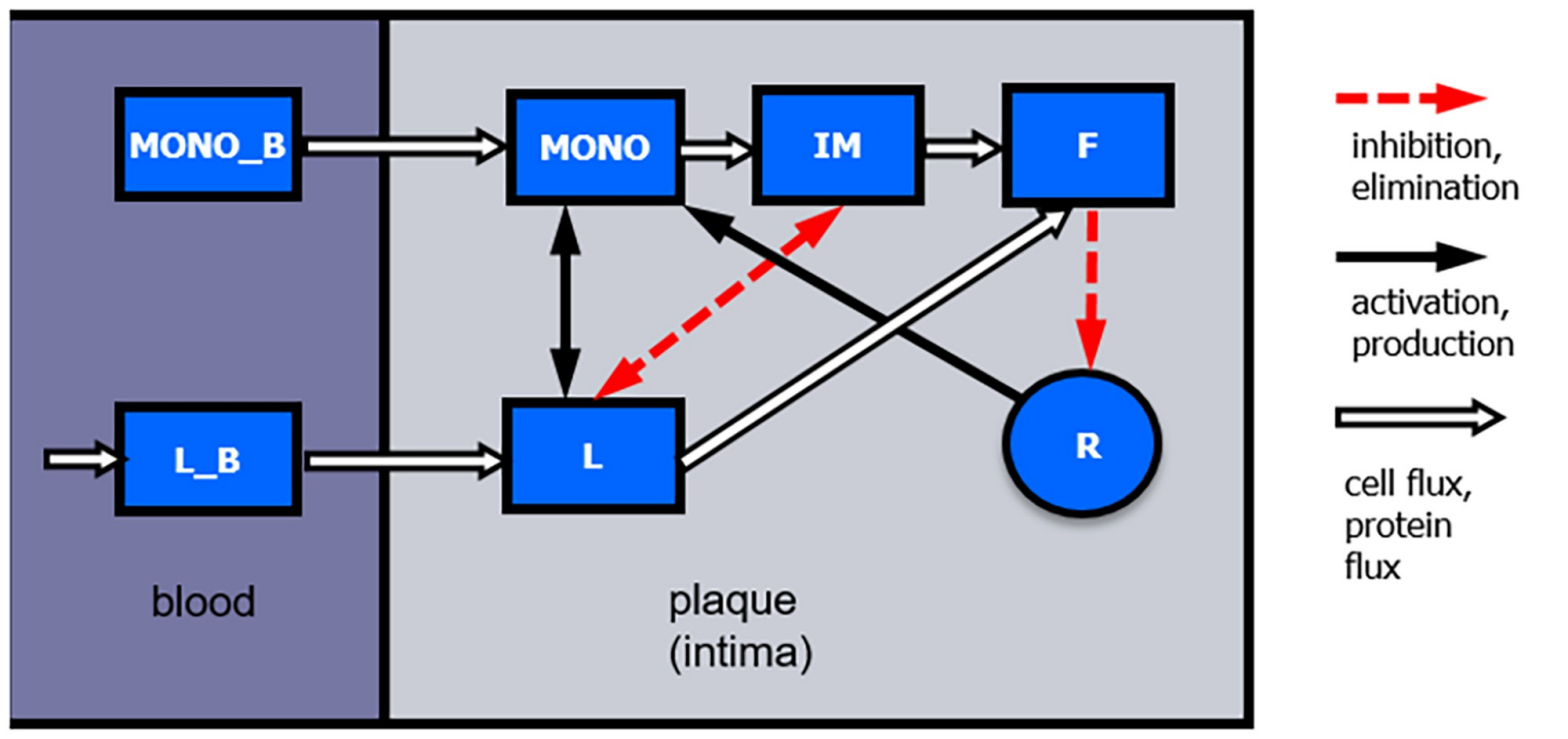
Structure of the model. The following compartments are considered: low density lipoprotein cholesterol (LDL-C) in blood (L_B_) and plaque (L), monocytes in blood (MONO_B_) and plaque (MONO), inflammatory macrophages in plaque (IM), foam cells in plaque (F) and the radius of the artery (R). Type and direction of arrows indicate fluxes or interactions.

We develop an ordinary differential equations model of atherosclerotic plaque formation in mice describing the dynamics of relevant lipid species and immune cells in blood and atherosclerotic lesions and the impact of interventions thereon. Moreover, we describe the dynamics of vessel occlusion. Major compartments and regulations of our model are presented in Fig 1. The model components and their relationship to biological quantities are summarized in Table 1.

**Table 1:** Variables.

model component	biological equivalent
R	radius of the vessel
F	foam cells
L	oxidized LDL-C in plaque
IM	inflammatory macrophages in plaque
MONO	monocytes in plaque
MONO_B_	blood monocytes
L_B_	blood LDL-C
lesion area (calculated from *R*)	lesion area

We consider normalized (dimensionless) model states throughout. For the purpose of comparisons with data, we multiply respective relative quantities with observed baseline values. All model equations are based on biologically motivated hypotheses translated into equations. We justify these assumptions and present derived equations in the following.

### Model equations and assumptions

Our model is based on the following biological assumptions: Endothelial dysfunction allows low density lipoprotein cholesterol (LDL-C) to migrate from blood into the intima. LDL-C particles in the intima are oxidized by monocytes that also migrate from blood into the lesion. These monocytes differentiate into macrophages, which consume the oxidized LDL-C particles and develop into plaque-forming foam cells. These foam cells result in a reduction of the radius of the artery [3, 14]. Thus, LDL-C levels in blood strongly affect plaque development in the intima. Lipid levels in blood are assumed to be dependent on diet and the three types of medication considered in the present paper, namely lipid lowering medication (propionic acid (PA)), antibiotics and immune-modulating therapy (IL-10 blockage).

We will derive respective equations in the following. Several of these equations were taken or adapted from the model of Bulelzai & Dubbeldam [14].

#### Monocytes in blood and plaque

We assume a constant production of blood monocytes and a first order degradation with rate *d*_MONO_B_. The efflux of monocytes from blood to intima is neglected in Eq 1.

$$\begin{array}{cc} \frac{d{\text{MONO}}_{B}}{dt} & =k_{\text{MONO}}-d_{\text{MONO}\_B}\cdot {\text{MONO}}_{B} \end{array}$$ (1)

The compartment MONO_B_ is normalized by assuming MONO_B__0_ = MONO_B_(0) = 1. To compare model and data, the output of MONO_B_ is multiplied by the value MONO_B__nor_, the average value of the control group of our experiments at the first measurement point.

We assume that blood monocytes enter the intima depending on LDL-C and the radius of the artery in a non-linear fashion. We follow [14] (eq. 3.3, 3.4) to model this dependency. Monocytes in intima are reduced by an efflux with rate *ϵ* and by differentiation into macrophages with rate *c*.

$$\begin{array}{c} \frac{d\text{MONO}}{dt}=\frac{a\cdot R^{3}}{R^{3}+\alpha }\cdot \frac{L}{1+L}\cdot {\text{MONO}}_{B}-\left(\epsilon +c\right)\cdot \text{MONO} \end{array}$$ (2)

According to [14] this equations is derived from the Navier-Stokes equation considering shear wall stress and assuming radial symmetry.

#### Inflammatory macrophages in plaque

If inflammatory macrophages take up (oxidized) LDL-C particles, they eventually transform into foam cells. Thus,

$$\begin{array}{cc} \frac{d\text{IM}}{dt} & =c\cdot \text{MONO}-b\cdot \text{IM}\cdot L \end{array}$$ (3)

where the second term represents the efflux into the foam cell compartment.

#### LDL (low density lipoprotein) cholesterol in blood and plaque

**Fig 2:**
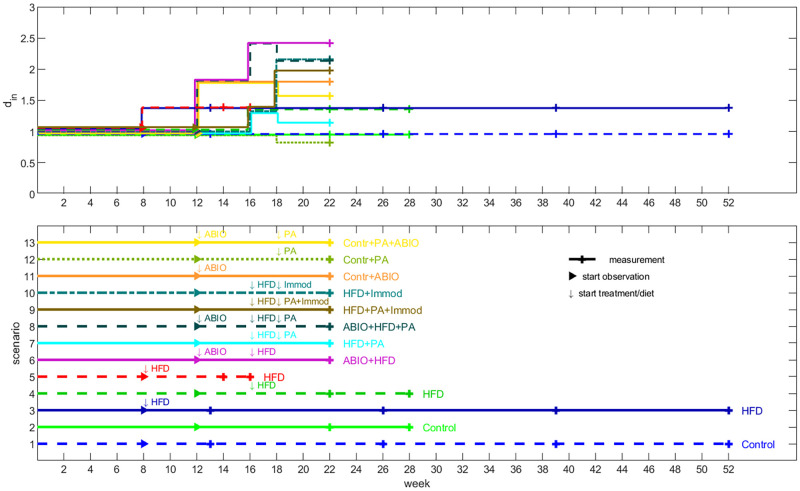
Experimental scenarios and respective step functions of LDL-C uptake. The upper diagram shows the step functions of LDL-C uptake *d*_*in*_ (we inserted small spaces between the lines to improve visibility of single lines), and the lower one represents the corresponding experimental settings for scenarios 1–13 (▶: start of observation, ↓: start of a medication or diet, −+−: measurement time point).

In our model, LDL-C in blood has a strong impact on atherosclerotic plaque formation. We assume a time-dependent uptake of LDL-C *d*_*in*_(*t*) from nutrition into blood, where *d*_*in*_(*t*) is a step function taking values of {1, *d*_ABIO_, *d*_Immod_, *d*_PA_, *d*_HFD_} and their products depending on the type of diet or intervention with the following notations: antibiotics: ABIO, immune modulating: Immod, lipid reducing: PA and high fat diet: HFD. The individual factors are multiplied if several of these interventions were applied at the same time (see S2 File). A value of *d*_*in*_(*t*) = 1 corresponds to normal diet without interventions. Fig 2 shows the functions *d*_*in*_ for the different experimental settings.

LDL-C is removed from blood with rate *d*_L__B_. The efflux of LDL-C from blood into intima is small compared to the LDL-C concentration in blood. Therefore, we neglect this part in Eq 4.

$$\begin{array}{c} \frac{dL_{B}}{dt}=d_{in}-{d_{L}}_{B}\cdot L_{B} \end{array}$$ (4)

The compartment L_B_ is normalized by setting L_B_(0) = L_B__0_ = 1. To compare model and data, L_B_ is multiplied by the value L_B__nor_, the average of control group samples taken at the first measurement point.

LDL-C entering the intima is assumed to be oxidized by monocytes present in the intima, i.e. in our model we do not distinguish between unoxidized and oxidized LDL-C. This process is assumed to be proportional to LDL-C in blood and a Michaelis-Menten term of monocytes indicating possible saturation. Thus, the term $k_{\text{LDL}}\cdot \frac{L_{B}\cdot \text{MONO}}{f+\text{MONO}}$ constitutes the uptake of (oxidized) LDL-C from blood according to [14]. Macrophages take up this LDL-C and convert into foam cells at rate *e*. We also assume an efflux of oxidized LDL-C from the plaque region [14] at rate *d*_LDL_, as discussed in [15].

$$\begin{array}{cc} \frac{dL}{dt} & =k_{\text{LDL}}\cdot \frac{L_{B}\cdot \text{MONO}}{f+\text{MONO}}-e\cdot L\cdot \text{IM}-d_{\text{LDL}}\cdot L \end{array}$$ (5)

#### Fat-laden foam cells in plaque

Foam cells are formed by the consumption of oxidized LDL-C by macrophages. Thus, the influx into this compartment equals the efflux from the macrophage compartment. Migration of foam cells or macrophages from lesions were observed [16, 17]. The underlying processes are complex and we assume for simplicity that foam cells migrate out of the plaque region at rate *d*_F_, similar to [18].

$$\begin{array}{cc} \frac{dF}{dt} & =b\cdot \text{IM}\cdot L-d_{F}\cdot F \end{array}$$ (6)

#### Radius of the artery and lesion area

The main outcome of the model is the vascular obstruction operationalized by a reduction of the radius R of the artery. We normalize R to one, i.e. *R*(0) = 1 for the lesion-free state. However, to start the process of plaque formation, it is necessary to assume a small initial lesion *R*(0) = *R*_0_ < 1. To describe the obstruction, we use the same geometric assumptions as in [14]. Thus, we assume axial symmetry, i.e. plaque deposits are rings on the vessel wall. Only foam cell dynamics contribute to radius reduction in our model:

$$\begin{array}{c} \frac{dR}{dt}=\frac{\xi }{2}\cdot (R-\frac{1}{R})\cdot \frac{dF}{dt} \end{array}$$ (7)

$$\begin{array}{cc} & =\frac{\xi }{2}\cdot (R-\frac{1}{R})\cdot \left(b\cdot \text{IM}\cdot L-d_{F}\cdot F\right) \end{array}$$ (8)

In our experimental data, lesion areas were determined. To compare this measured quantity with our model outputs, we calculated the lesion area from the radius via *π*⋅(1−*R*^2^).

### Numerical methods for simulation

Differential equations are implemented in MATLAB 9.6.0.1072779 (R2019a) using the SIMULINK toolbox (The MathWorks Inc., Natick, MA, USA). Numerical solutions of the differential equation system are obtained using the variable step solver from Adams and Bashford (ode113, SIMULINK toolbox) with an absolute and a relative tolerance of 10^−10^. Fixed step solvers also yield to good results as long as the time step size is below 0.1d.

### Data

Model simulations are compared with data from literature or own mice experiments comprising time series of lesion area, LDL-C and monocytes in blood. Experimental settings comprise high vs. normal fat diet and treatments with antibiotics, immune-modulating therapy (IL-10 blockage), lipid lowering medication (PA) and combinations of it. Experimental groups are displayed in Table 2.

**Table 2:** Experimental groups. Groups differ with respect to diet (high fat diet vs. normal diet), antibiotic treatment (broad-spectrum antibiotics vs. none), immune modulation (IL-10 blockage or not) and lipid lowering agent (short-chain fatty acid propionate vs. none). Data of scenarios 1, 3 and 5 were retrieved from the literature.

diet	antibiotics	lipid lowering	immune therapy	ref.	scenario
normal	–	–	–	[21]	1
normal	–	–	–		2
high fat	–	–	–	[21]	3
high fat	–	–	–		4
high fat	–	–	–	[22]	5
high fat	+	–	–		6
high fat	–	+	–		7
high fat	+	+	–		8
high fat	–	+	+		9
high fat	–	–	+		10
normal	+	–	–		11
normal	–	+	–		12
normal	+	+	–		13

The automated tool “ycasd” [19] was used to extract literature data from published figures as precisely as possible. S2 File contains the data used.

All animals used in our own experiments were bred, raised, and housed in the “Forschungseinrichtungen für Experimentelle Medizin” (FEM, Charité—University Medicine Berlin, Germany) facilities under specific pathogen-free (SPF) conditions. All experiments were in accordance with the German/European law for animal protection and were approved by the local ethics committee (Landesamt für Gesundheit und Soziales Berlin, G0295/16). Mice had constant access to food and water and were maintained on a 12h/12h day/night cycle. Adult (16 weeks of age) female C57BL/6J, APOE-/- (Charles River) were age-matched and randomly assigned to either a standard chow diet (SCD: crude fat 4.1%, cholesterol 14 mg/kg; Ssniff, Soest, Germany, E15000) or a high-fat diet (HFD: crude fat 34.6%, cholesterol 290 mg/kg; Ssniff, Soest, Germany, E1574) for a total of 6 or 12 weeks. Mice were continuously kept in a sterile environment (autoclaved food and drinking water, sterile filtered antibiotic cocktail) and handled under strict aseptic conditions to avoid contamination.

Mice were treated with the above mentioned options. In detail, antibiotic treatment comprised a quintuple treatment applied for six weeks, namely ampicillin plus sulbactam (1 g/L), vancomycin (500 mg/L), ciprofloxacin (200 mg/L), imipenem (250 mg/L) and metronidazole (1 g/L) in the drinking water resulting in a complete eradication of intestinal microbiota. The short-chain fatty acid propionate (PA), calcium propionate (150 mM, Sigma-Aldrich) was used as lipid-lowering medication. PA was administered daily via oral gavage for four weeks starting after two weeks of the respective diet. Since the lipid lowering effect of PA depend on modulation of the intestinal immune system involving IL-10 receptor signaling, we blocked IL-10 receptor pathway by intraperitoneal injection of an anti-IL-10 receptor monoclonal antibody (clone 1B1.2, 1mg per mouse per week, n = 8) for four weeks starting after two weeks of the respective diet to validate the regulatory property of IL-10 receptor signalling on lipid metabolism. This blockage is supposed to increase LDL-C concentrations by upregulation of the Niemann-Pick C1-like 1 gene. Further details of the experiments are described in [20].

After 6 or 12 weeks with the respective diet and treatments, all mice were sacrificed by cervical dislocation to collect blood and organs. Following sacrifice, basal segments of the mouse heart were immediately embedded in tissue-freezing medium (Leica) and frozen on dry ice. The tissues were stored at -80°C until further use. Sections of the aortic root (5 *μ*m) were prepared on glass slides (Thermo Scientific, Super frost PLUS) using a Cryostat Microtome (Microm HM 560) and stained with Oil Red O to quantify atherosclerotic burden.

For lipoprotein measurements, plasma samples were analysed by fast-performance liquid chromatography (gel filtration on Superose 6 column (GE Healthcare)). Different lipoprotein fractions were separated and evaluated based on flow-through time. Cholesterol levels were measured by an enzymatic assay (Cobas, Roche) according to the manufacturer’s protocol.

### Estimation of parameters

We determined the parameter values by optimizing the agreement of simulation results and data using the following goal function.

$$\begin{array}{c} \sum_{j=1}^{L}{\left(\frac{f_{\text{model}}\left(t_{j},k\right)-f_{\text{data}}\left(t_{j}\right)}{\sigma_{j}}\right)}^{2}\to \underset{k}{\text{min}}, \end{array}$$ (9)

The solution of the differential equation system with the parameter set **k** = *k*_1_, …*k*_*n*_ at time points *t*_*j*_ is represented by *f*_*model*_(*t*, **k**), and *f*_*data*_(*t*_*j*_) are means of measurements at a certain time point of an experimental group with standard deviation *σ*_*j*_. The vector **k** corresponds to all parameters included in the fitting procedure. In the following, equation (9) is called fitness function. Simultaneous optimization of several scenarios is achieved by adding the respective fitness functions. As in our previous work [23], we solved the optimization problem using (1+3)-evolutionary-strategies with self-adapting mutation step size (see [24, 25]).

To compute 95% confidence intervals of our parameter estimates, we use the method proposed in [26, 27]. In brief, for each time point with measurements, we created a virtual set of random data points using mean and variance of the existing data. Then, the model was fitted to the virtual data with the method described above. We used 1000 repetitions to determine the confidence ranges for our parameters.

To determine parameter sensitivity, we changed individual parameters by +/- 1% leaving the other parameters unchanged. The associated deterioration of the fitness function was then evaluated.

The second time points of scenarios 2 and 4 (week 28, see Table 2) were not included in the fitting and was used for validation of model predictions.

## Results

### Parameter estimates

The fitting procedure included 11 parameters without therapy, the initial radius of artery *R*_0_ and four intervention-related parameters. Due to low identifiability or normalization, the parameters *d*_MONO__B_, *k*_MONO_ and *d*_L__B_ were excluded from fitting and set to one.

Parameter estimates and initial values are presented in S1, S2 Tables in S1 File. We mostly consider dimensionless parameters expect for the baseline values of blood LDL-C and monocytes, which are multiplied with the respective model outputs to allow comparisons of model and data.

**Fig 3:**
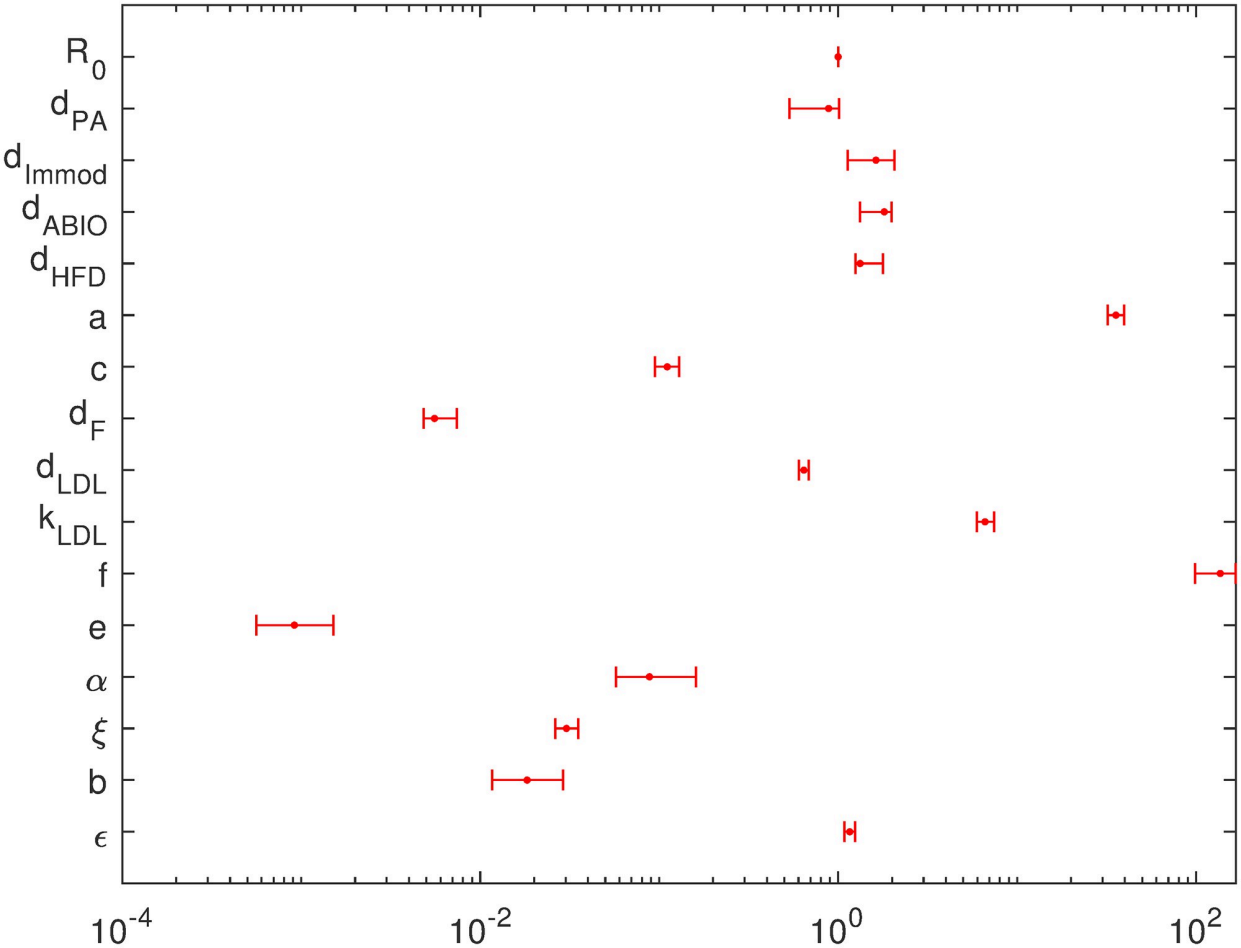
Confidence intervals of parameter estimates. Error bars show model parameters and corresponding 95% confidence intervals.

Empirical confidence intervals of parameter estimates are shown in Fig 3. Results of sensitivity analysis are shown in the S1 Fig in S1 File. Parameters showed a reasonable identifiability.

The different experiments from Table 2 are modelled by estimating the steps of the function *d*_*in*_. Experimental settings and resulting step functions *d*_*in*_ are shown in Fig 2. Step parameters are provided in S1 Table in S1 File. Although no prior constrains were applied, estimated step parameters reflect the expected impact on LDL-C uptake, i.e. step parameters of high-fat diet, antibiotics and IL-10 blockage were larger than one while that of propionic acid treatment was smaller than one [20].

### General model behaviour

**Fig 4:**
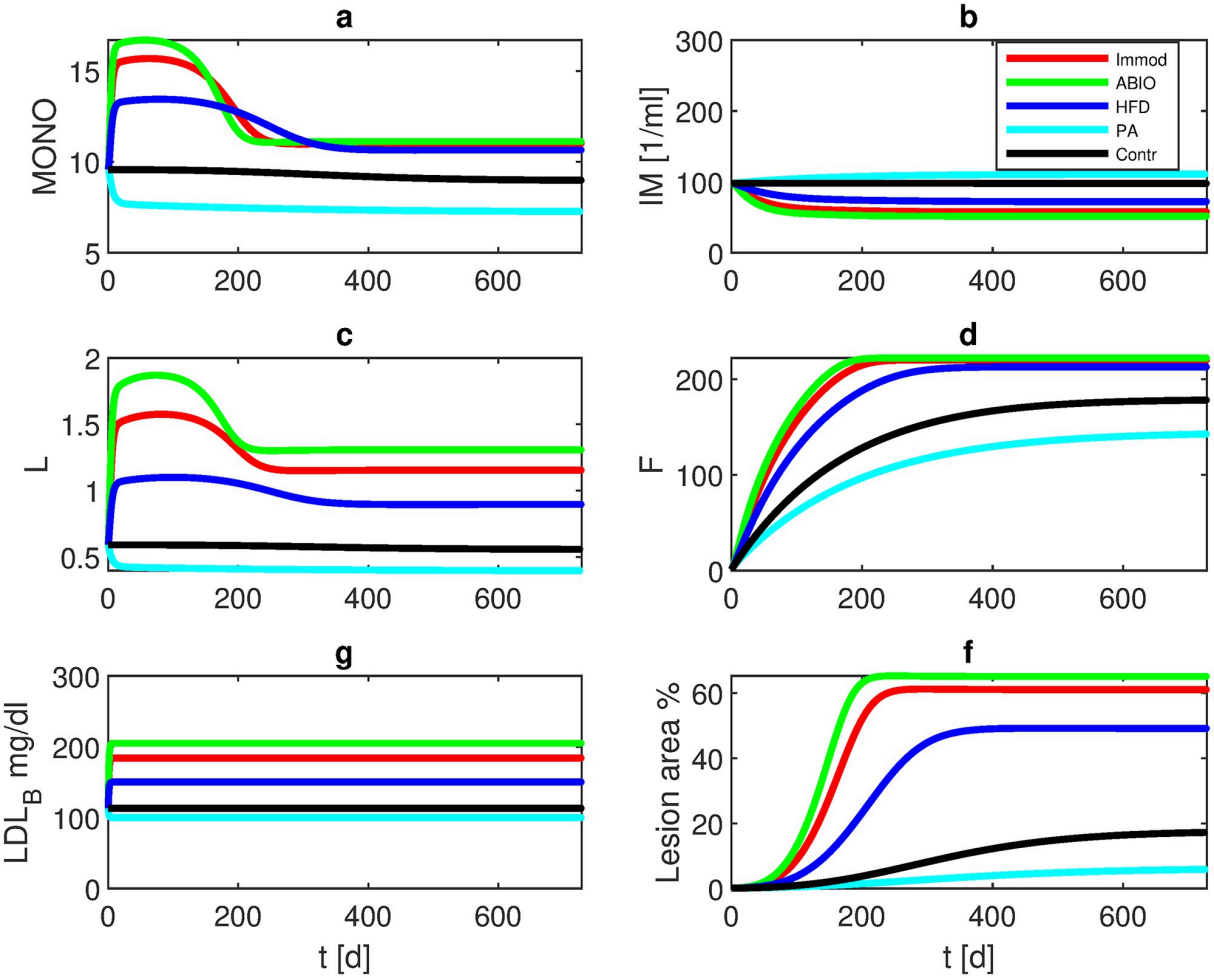
Long term simulation of different single interventions. Simulation is started with *R*_0_ = 0.9996. Over a period of two years, normal diet (Contr), high fat diet (HFD), immune modulating agents (IMMOD), antibiotic drugs (ABIO), or lipid lowering agent (PA) are simulated. The curves represent model results of a) monocytes in plaque, b) macrophages in plaque, c) LDL-C in plaque, d) foam cells, e) LDL-C in blood, and f) lesion area. Different steady-states are established. Respective values are provided in S3 Table in S1 File.

Analysis of the model behaviour reveals an unstable trivial steady-state of no lesion (*R*(*t*) = 1). Thus, to initiate plaque formation, a small initial lesion *R*_0_ < 1 is required. Dependent on the initial settings, the model converges to a new steady-state. Starting from a state configuration of normal initial values except for a small initial lesion of *R*_0_ = 0.9996, unique steady-states are established which differ for the different experimental settings as shown in Fig 4. Steady-state values for the single interventions are provided in S3 Table in S1 File.

Comparing the single interventions, antibiotic treatment resulted in the largest lesion area (65%) followed by IL-10 blockage (61%) and high-fat diet (49%). The control group stabilizes at 17% luminal reduction, while propionic acid treatment ameliorates atherosclerosis formation (6% lesion area) (see Fig 4 and S3 Table in S1 File).

### Comparison of model and data

**Fig 5:**
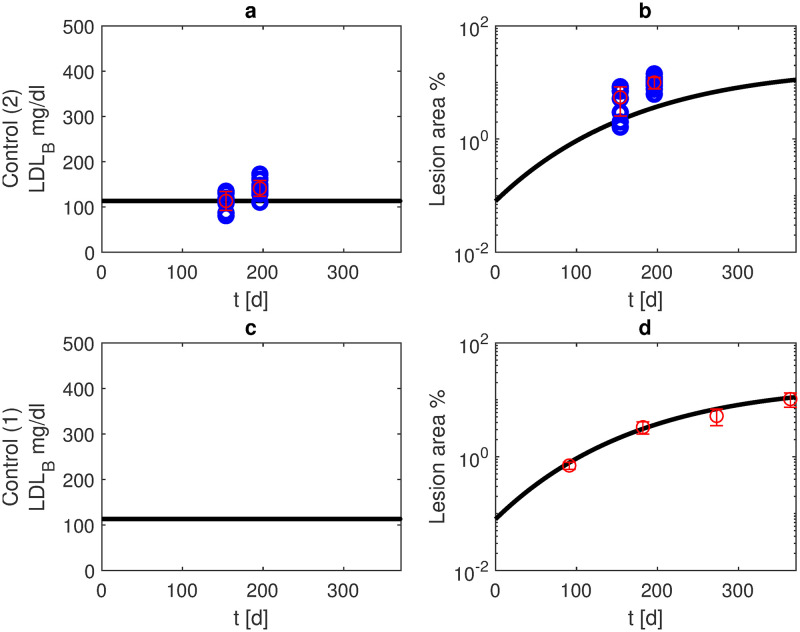
Normal diet. We present model simulations and data of APOE -/- mice under normal diet (blue: data points, red: mean and standard deviation for scenarios 2 (panels a, b, own experiments) and 1 (panels c, d, data from [21]). Black curves represent model simulations of LDL-C in blood (panels a, c) and lesion area (panels b, d).

For the majority of scenarios and time points, parameter estimates resulted in a good agreement of model and data. Under normal diet, APOE -/- mice develop atherosclerosis [21, 28], gradually worsening over the lifespan. Our model simulations predict a lesion area of about 3% after six months and about 11% after one year (see Fig 5).

**Fig 6:**
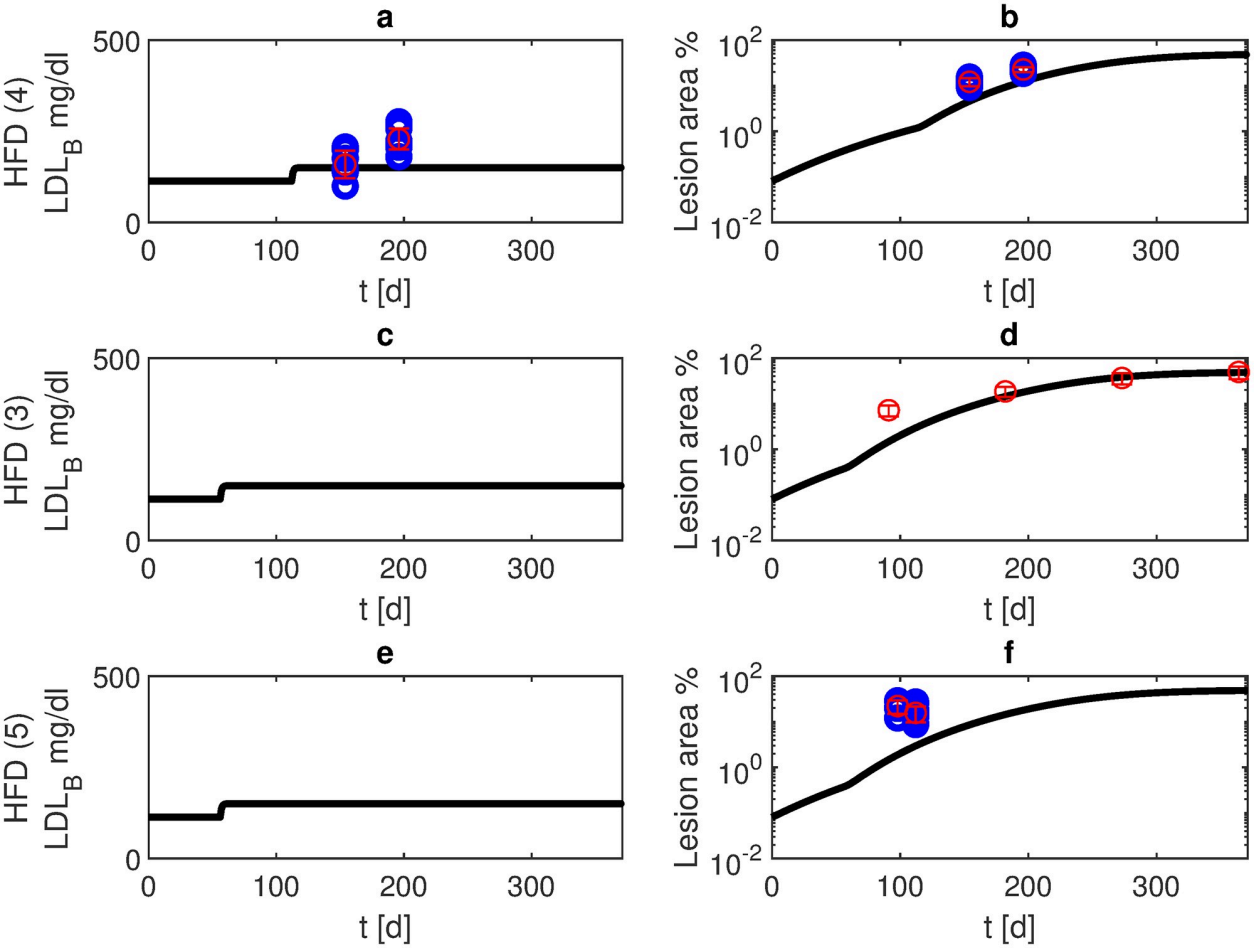
High fat diet. We present model simulations and data of APOE -/- mice at high fat diet (blue: data points, red: mean and standard deviation for scenarios 4 (panels a, b, own experiments), 3 (panels c, d, data from [21]), and 5 (panels e, f, data from [22]). Black curves represent model simulations of LDL-C in blood (panels a, c, e) and lesion area (panels b, d, f). In scenario 4, high fat diet started at the age of 16 weeks while in scenarios 3 and 5 it started at the age of eight weeks.

At HFD, lesion development increases significantly [21]. In scenarios 3 and 5 starting high fat diet at the age of eight weeks, model simulations result in about 11% volume reduction after six months and 48% after one year (Fig 6). In scenario 4 starting the diet at the age of 16 weeks, simulations result in about 9% volume reduction at six months and 48% after one year.

**Fig 7:**
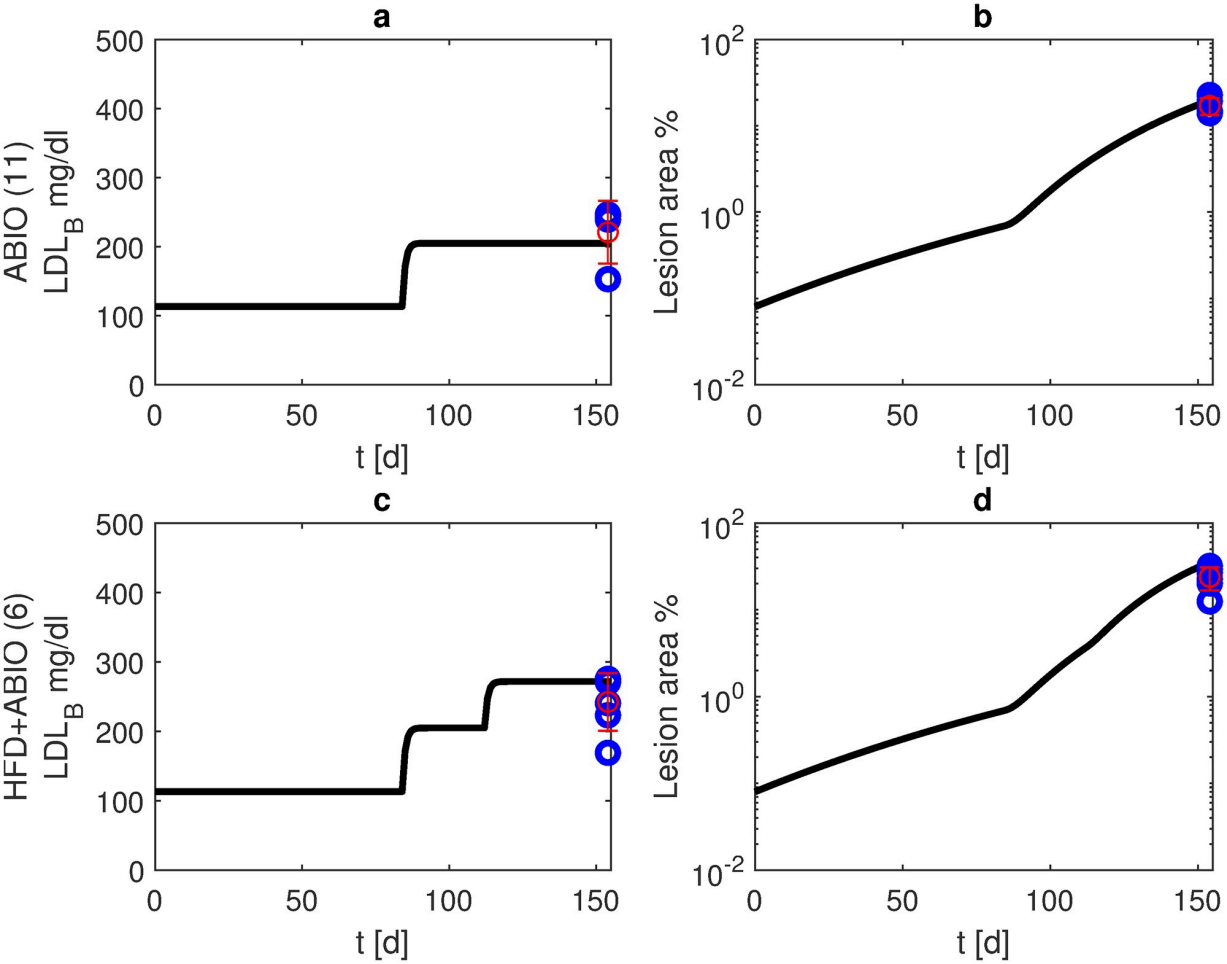
Antibiotic treatment. We present simulation results and data from own experiments of APOE -/- mice receiving antibiotics from the age of 12 weeks until the end of observation period (blue: data points, red: mean and standard deviation for scenarios 11 (normal diet, panels a, b) and 6 (HFD starting at week 16, panels c, d)). Black curves represent model simulations of LDL-C in blood (panels a, c) and lesion area (panels b, d).

Antibiotics can reduce the growth of inflammation- or infection-related atherosclerotic plaques [29]. On the other hand, antibiotics destroy the intestinal flora, and thus, change lipid absorption. Model simulations of normal diet combined with antibiotic therapy resulted in a lesion area of about 38% after six months, more than ten times the value of normal diet without antibiotics (Fig 7, scenario 11). If HFD is combined with antibiotics, lesion formation is further worsened with about 64% after six months (Fig 7, scenario 6).

**Fig 8:**
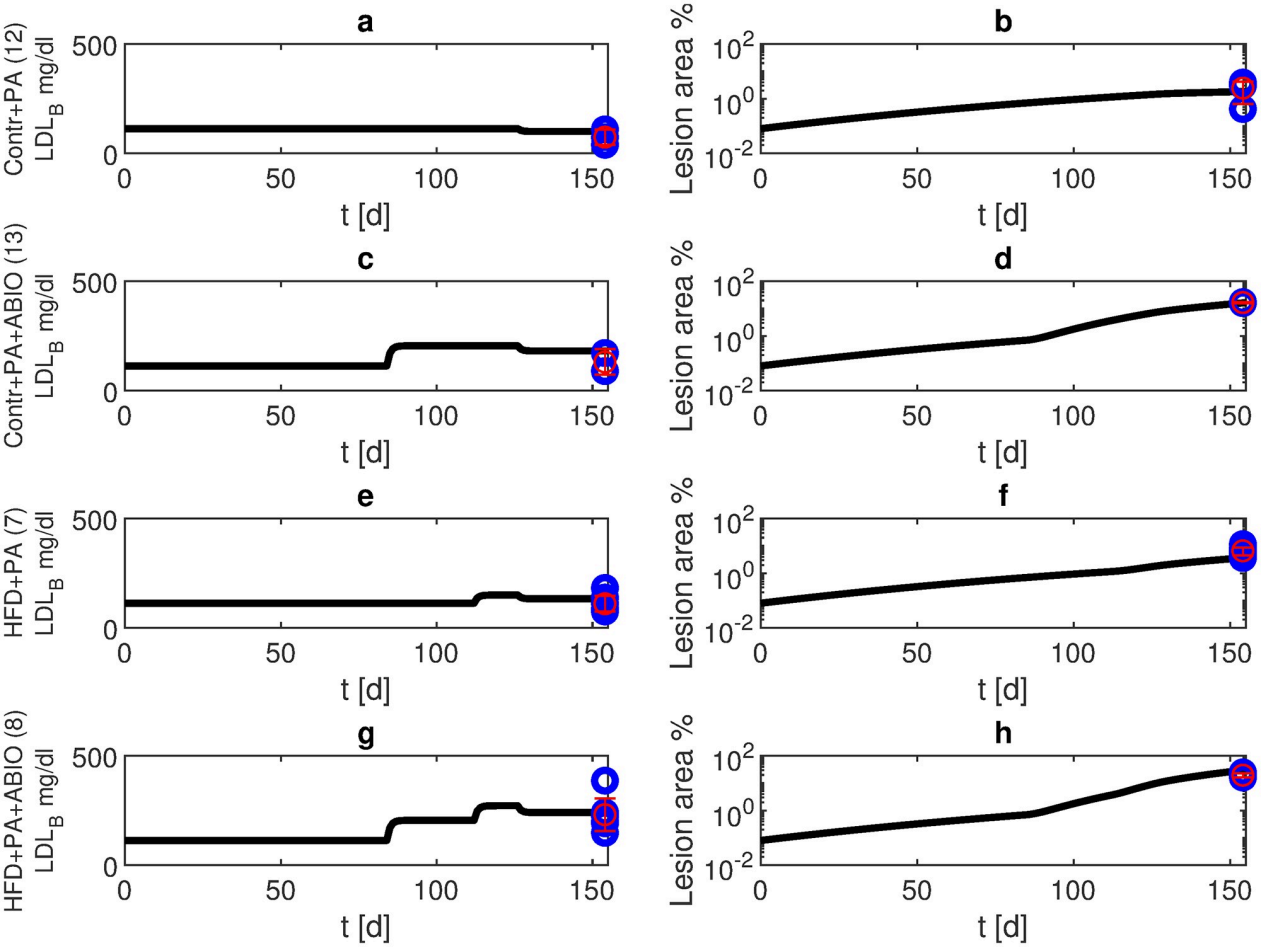
Propionic acid treatment. We present simulation results and data from own experiments of APOE -/- mice receiving propionic acid from the age of 18 weeks until the end of observation period (blue: data points, red: mean and standard deviation for scenarios 12 (normal diet, panels a, b), 13 (antibiotic treatment from week 12, panels c, d), 7 (HFD from week 16, panels e, f), and 8 (antibiotic treatment from week 12 and high fat diet from week 16, panels g, h). Black curves represent model simulations of LDL-C in blood (panels a, c, e, g) and lesion area (panels b, d, f, h).

Lipid-lowering drugs are used for prevention and therapy of dyslipidemia induced atherosclerosis resulting in reduction of major vascular events [30]. In our experiments of mice under normal diet, lipid lowering therapy with PA started at week 18. As a result, after six months only 2% of the blood vessel was occluded in our model simulations in agreement with the data (Fig 8, scenario 12). If applied in combination with antibiotics, PA can ameliorate lesion formation. In our experiments, antibiotic therapy was given from week 12, while lipid lowering prevention again started at week 18. Resulting lesions were smaller than with antibiotics alone. Our model predicts a 28% volume reduction after six months in agreement with the data (Fig 8, scenario 13). The effect of high fat diet starting at the age of 16 weeks can also be ameliorated by PA. After six months, the lesion area was about 6% (see Fig 8, scenario 7), i.e. smaller than in the group with high fat diet alone. Next we considered a combined effect of antibiotics starting at week 12, high fat diet starting at week 16, and PA starting at week 18. After six months, the lesion area was about 54% (see Fig 8, scenario 8).

**Fig 9:**
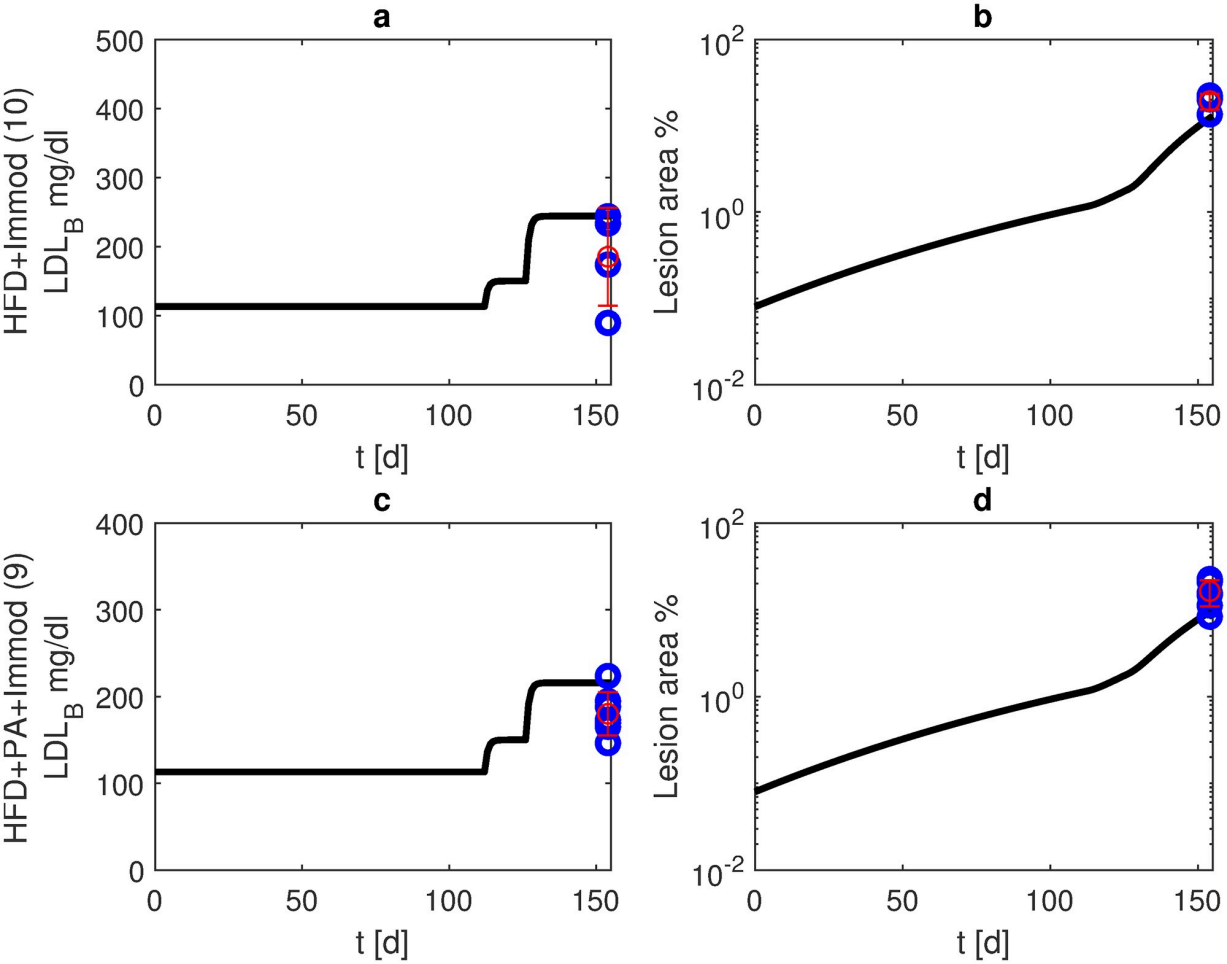
Immune modulating therapy and HFD with or without PA treatment. We present model simulations and data of APOE -/- mice under HFD starting at the age of 16 weeks and IL-10 blockage starting at the age of 18 weeks until the end of observation period (blue: data points, red: mean and standard deviation for scenarios 10 (without PA, panels a, b) and 9 (with PA from week 18, panels c, d). Black curves represent model simulations of LDL-C in blood (panels a, c) and lesion area (panels, b, d).

Finally, we combined HFD and immune modulating medication with or without PA treatment. Without PA (scenario 10), lesions grew to 37% after six months, while with additional PA treatment (scenario 9), lesions were significantly lower with 27% (Fig 9).

### Model predictions

**Fig 10:**
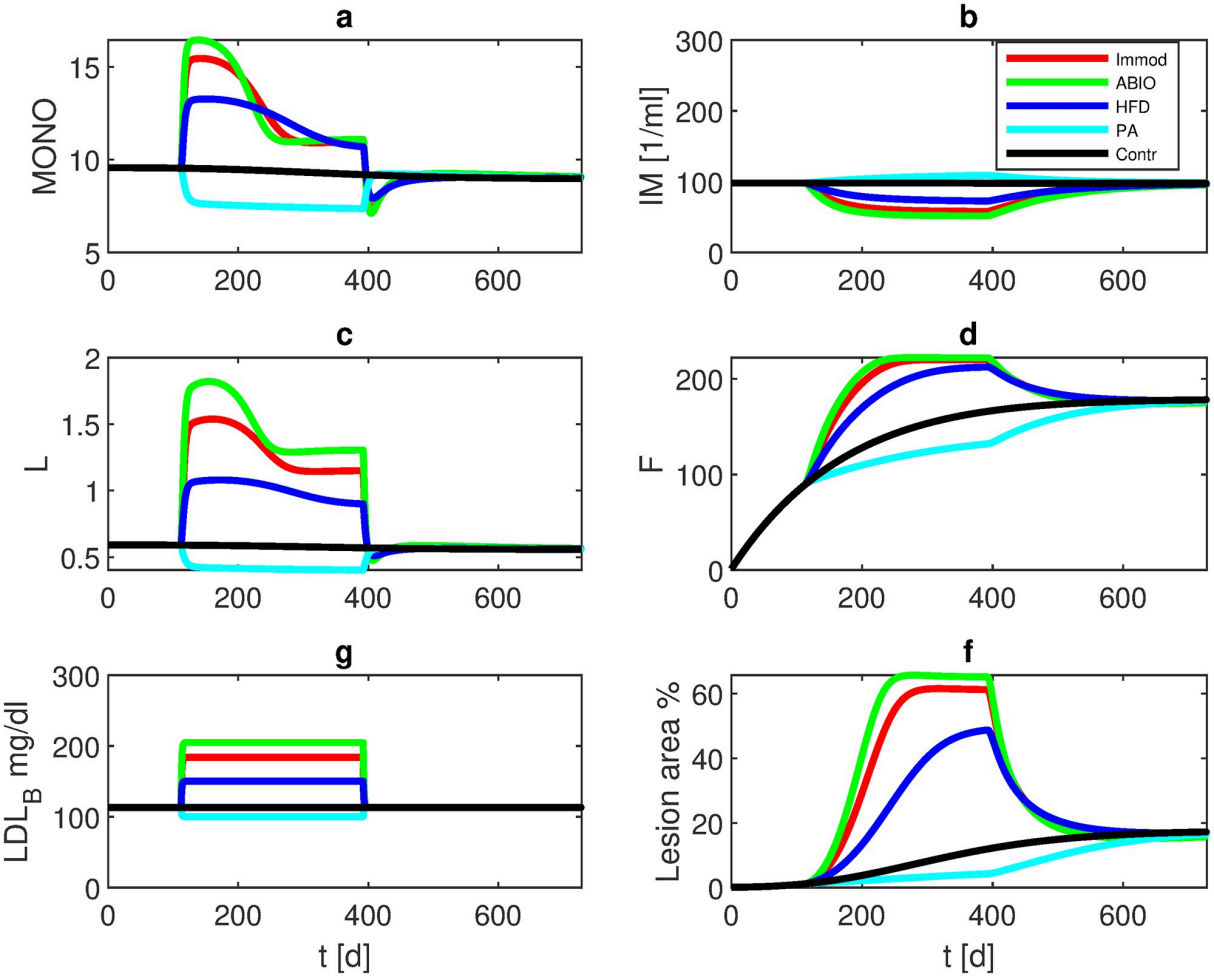
Regeneration after stopping interventions. We simulated mice with normal diet and without medication until week 16. From week 16 until week 56, HFD, immune modulation, antibiotics, or PA are given, followed by normal diet without therapy again. The curves represent model results of a) monocytes in plaque, b) macrophages in plaque, c) LDL-C in plaque, d) foam cells, e) LDL-C in blood, and f) lesion area. It revealed that the interventions are reversible, i.e. the system returns to the trajectory of the control group.

Our model can be used to make predictions of scenarios which are not yet tested experimentally. We first examined how the system behaves after stopping diet or medication interventions. Our simulation starts with a normal diet up to week 16. After that, HFD, immune-modulating therapy, antibiotics or PA are given for 40 weeks. From week 56, normal diet is applied again, without medications. Over the course of the next two years, the system returns to a state that would have been achieved with normal diet without interventions (see Fig 10), i.e. the interventions turned out to be reversible in our model.

**Fig 11:**
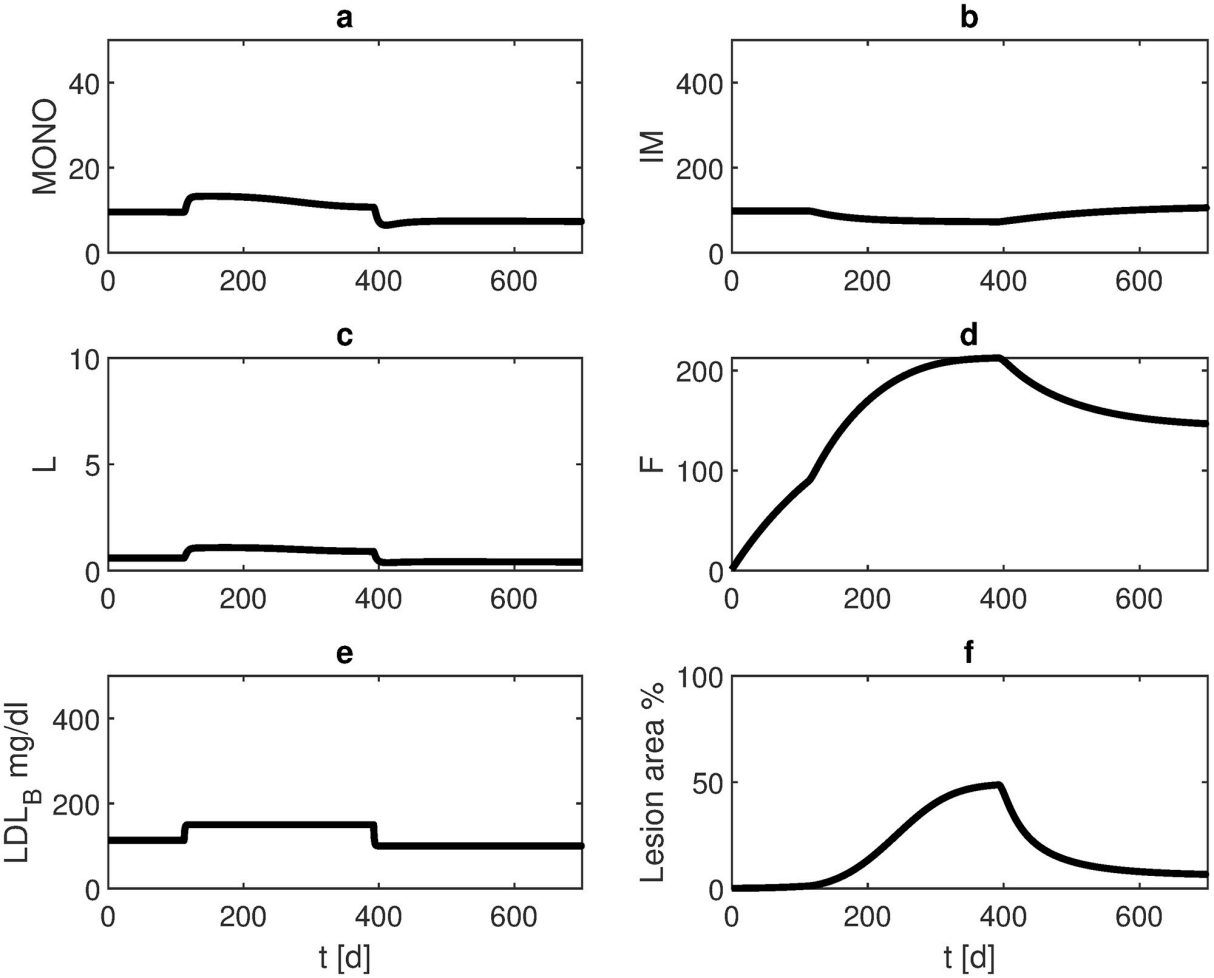
Propionic acid after high fat diet. HFD is given from week 16 until week 56. PA is applied between week 57 and week 100. The curves represent model results of a) monocytes in plaque, b) macrophages in plaque, c) LDL-C in plaque, d) foam cells, e) LDL-C in blood, and f) lesion area. The lesion regresses under lipid therapy.

Finally, we studied whether the lesion size after HFD could be reduced by PA. High fat diet is given from week 16 until week 56 resulting in a large lesion with 47% of vascular occlusion. After returning to normal diet, PA is applied. At week 100, the lesion was reduced to about 3% (Fig 11).

## Discussion

Understanding the mechanisms of atherosclerosis is important for better prevention and therapy. We here propose a model of atherosclerotic plaque formation in mice including different types of interventions like diet, immune-modulating therapy, propionic acid treatment, and antibiotics. The model was parametrized on the basis of experimental data from APOE -/- mice taken from literature and our own experiments. Model parametrization resulted in a good agreement of model predictions and data and a well-identifiable set of parameters. The model allows for simulation of yet untested therapy schedules.

Our model is based on a model proposed by [14]. It describes the interaction of monocytes, macrophages, oxidized LDL-C, and foam cells shrinking the radius of the artery dependent on wall shear stress. We adapt this model by adding the influx of monocytes into the plaque region from an additional peripheral blood compartment. Concentration of LDL-C in peripheral blood is also considered. On the other hand, we simplified the model of vascular obstruction compared to [18] by dropping HDL-C as a protective component due to lack of evidence. To investigate the effects of diet and medication on atherosclerosis, we include the effects of antibiotics, immuno-modulation and propionic acid treatment. All interventions have an impact on blood LDL-C concentrations. Of note, our respective parameter estimates of our model correctly reflects the supposed impacts on LDL-C levels.

We developed our model on the basis of data from APOE -/- mice which develop atherosclerosis without interventions. In [28], small lesions are detected already at the age of 4 and 10 weeks. Therefore, we assume that murine plaque formation begins immediately from birth, and thus, in our model plaque size starts growing at t = 0 with a small initial lesion.

Growth and regression of plaques, dependent on LDL-C and HDL-C concentrations in peripheral blood with additional consideration of oxidative stress or antioxidant deficiency, high blood pressure and cigarette smoking as risk factors was modelled in [7, 8]. Using partial differential equations, the authors describe the cross section of an artery, cross sections along the artery and plaque weight. Results were compared to mice data. The influence of cytokines, growth factors and migration of smooth muscle cells on intima thickening is analysed in partial differential equations models proposed in [9, 10], and model results were compared with data from rabbits. Another partial differential equations model [11] considers macrophages, necrotic cells, oxidized lipids, oxygen concentration, and platelet derived growth factor and describes vessel growth. This model was created on the basis of serial ultrasound images. Regression of plaques under antioxidants is examined in ODE and PDE models proposed in [12]. Atherosclerosis under statin medication is studied in the modelling work of [13]. A partial differential equations model to reproduce atheroma plaque growth in coronary arteries is proposed in [31]. The model includes LDL-C, oxidized LDL-C, monocytes, macrophages, foam cells, smooth muscle cells, cytokines and collagen. Most of these models were not parametrized on large sets of experimental data.

Compared to these approaches, we parameterize our model on the basis of a larger data base comprising several types of interventions. On the other hand, only a few measurements over time were available for both, the literature data and our own experimental data as well. Measurements were available only for a few state parameters of the model such as monocytes, LDL-C in blood and lesion area. In some instances, values of only two animals were available. This further limits the complexity of an atherosclerosis model which could be parametrized on the basis of the available sparse data. Thus, it was necessary to consider a simplified model. In particular, we focused our modelling on quantifiable parameters and did not include, for example, smooth muscle cells which would require complex mechanistic assumptions and single-cell data. We also did not consider inflammatory effects, an issue we aim to improve in later model versions and experiments. This simplification could be a reason of the less optimal fit of scenarios with HFD known to induce low-grade inflammation. Processes involved in advanced stages of atherosclerosis, such as increasing numbers of Ly6Chi monocytes in the blood [32, 33], were also not yet modelled. Our model is intended to describe atherosclerosis development at an early stage, i.e. the deposition of plaques in the vessel walls is studied before advanced plaque morphology occurred. Finally, the spatial form of the plaques, as represented e.g. in PDE models [7, 8, 10, 31] was not considered due to lack of sufficiently detailed data. Denser time resolution in atherosclerosis experiments and more detailed readouts are required to allow parametrization of more complex models. On the other hand, our model provides testable hypotheses which could be validated in future experiments such as those provided in the prediction section.

Asymmetric or outward vascular remodeling plays an important role in atherosclerosis development in humans [18, 34]. However, in our experimental images in mice we predominantly observed inward plaque development. To illustrate this phenomenon, we provide representative histological images of atherosclerotic plaques in HFD fed APOE -/- mice after six weeks as compared to mice treated with propionic acid and HFD (S2 Fig in S1 File). This qualitative difference needs to be considered when translating the model to the human situation, which we plan for the future.

In conclusion, we proposed a biomathematical model of atherosclerosis development in APOE -/- mice including a well-defined set of physiological parameters. Different diet- and treatment schedules are considered and their effects on atherosclerosis development were quantified based on experimental data. We demonstrated how the model could be used to predict the atherosclerotic effect of new combinations of these therapies. We plan to extend our model in the future, in particular by taking infection-induced inflammation and resulting progression of atherosclerosis into account.

## Supporting information

S1 File A biomathematical model of atherosclerosis in mice—supplement material. The file contains estimated model parameters, results of sensitivity analyses, estimated steady-states after interventions and plaque images.
(PDF)

S2 File A biomathematical model of atherosclerosis in mice—Data used for model development. The file contains data used for modelling.
(XLSX)
