# Supplementary material for: A biomathematical model of atherosclerosis in mice
Source: PLoS One. 2022 Aug 3;17(8):e0272079. doi: 10.1371/journal.pone.0272079 (PMC9348695; doi:10.1371/journal.pone.0272079)
Supplement: S1 File — The file contains estimated model parameters, results of sensitivity analyses, estimated steady-states after interventions and plaque images. (PDF) [file pone.0272079.s001.pdf]

# A biomathematical model of atherosclerosis in mice – supplement material

Sibylle Schirm[1*], Arash Haghikia[2,3,7], Markus Brack[4,5], Peter Ahnert[1], Geraldine Nouailles[4], Norbert Suttorp[5], Markus Loeffler[1], Martin Witzenrath[4,5], Markus Scholz[1,6*]

**1** Institute for Medical Informatics, Statistics and Epidemiology, University of Leipzig, Leipzig, Germany
**2** Department of Cardiology, Charité – Universitätsmedizin Berlin, Campus Benjamin Franklin, Berlin, Germany
**3** DZHK (German Center for Cardiovascular Research), partner site Berlin, Berlin, Germany
**4** Charité – Universitätsmedizin Berlin, Corporate Member of Freie Universität Berlin and Humboldt-Universität zu Berlin, Division of Pulmonary Inflammation, Berlin, Germany
**5** Charité – Universitätsmedizin Berlin, Corporate Member of Freie Universität Berlin and Humboldt-Universität zu Berlin, Department of Infectious Diseases and Respiratory Medicine, Berlin, Germany
**6** LIFE Research Center of Civilization Diseases, University of Leipzig, Leipzig, Germany
**7** Berlin Institute of Health (BIH), Berlin, Germany

* sibylle.schirm@uni-leipzig.de

## Parameters

**Table S1. Parameters.**

| compartments *Plaque* and *Blood* | | | | |
|---|---|---|---|---|
| parameter | meaning | value | 95% confidence intervals | |
| $\epsilon$ | monocyte diffusion | 1.160E+00 | [ 1.083E+00 , 1.245E+00 ] | fitted [1] |
| $b$ | rate of L ingestion by IM | 1.828E-02 | [ 1.168E-02 , 2.906E-02 ] | fitted [1] |
| $\xi$ | foam cell volume factor | 3.031E-02 | [ 2.631E-02 , 3.528E-02 ] | fitted [1] |
| $\alpha$ | flow parameter | 8.816E-02 | [ 5.732E-02 , 1.602E-01 ] | fitted [1] |
| $e$ | rate of L ingestion by IM | 9.150E-04 | [ 5.595E-04 , 1.515E-03 ] | fitted [1] |
| $f$ | LDL-uptake and oxidation by mono is saturated | 1.365E+02 | [ 9.863E+01 , 1.667E+02 ] | fitted [1] |
| $k_{\mathrm{LDL}}$ | LDL transfer rate into intima | 6.618E+00 | [ 5.965E+00 , 7.444E+00 ] | fitted |
| $d_{\mathrm{LDL}}$ | LDL diffusion out of the plaque region | 6.431E-01 | [ 6.017E-01 , 6.829E-01 ] | fitted [1] |
| $d_{\mathrm{F}}$ | foam cell migration rate | 5.549E-03 | [ 4.821E-03 , 7.400E-03 ] | fitted [2] |
| $c$ | differentiation of monocytes into macrophages | 1.108E-01 | [ 9.453E-02 , 1.291E-01 ] | fitted [1] |
| $a$ | rate at which monocytes enter the intima | 3.562E+01 | [ 3.210E+01 , 3.958E+01 ] | fitted [1] |
| $d_{\mathrm{HFD}}$ | factor of LDL-C uptake during HFD | 1.326E+00 | [ 1.250E+00 , 1.776E+00 ] | fitted |
| $d_{\mathrm{ABIO}}$ | factor of LDL-C uptake during antibiotic therapy | 1.810E+00 | [ 1.321E+00 , 1.992E+00 ] | fitted |
| $d_{\mathrm{Imsupp}}$ | factor of LDL-C uptake during immuno-modulating therapy | 1.626E+00 | [ 1.130E+00 , 2.063E+00 ] | fitted |
| $d_{\mathrm{PA}}$ | factor of LDL-C uptake during propionic acid treatment | 8.845E-01 | [ 5.350E-01 , 1.009E+00 ] | fitted |
| $d_{\mathrm{MONOB}}$ | blood monocyte degradation | 1.000E+00 | | set |
| $k_{\mathrm{MONO}}$ | blood monocyte influx | 1.000E+00 | | set |
| $d_{\mathrm{LB}}$ | LDL-C removal from blood | 1.000E+00 | | set |

**Table S2. Initial and normal values.**

| parameter | meaning | value | 95% confidence intervals | |
|---|---|---|---|---|
| $\mathrm{R}_0$ | initial artery radius | 9.996E-01 | [ 9.992E-01 , 9.998E-01 ] | fitted |
| $\mathrm{F}_0$ | foam cells | 1.000E+00 | | set |
| $\mathrm{L}_0$ | oxidized LDL-C in plaque | 5.919E-01 | | set |
| $\mathrm{IM}_0$ | inflammatory macrophages in plaque | 9.808E+01 | | set |
| $\mathrm{MONO}_0$ | monocytes in plaque | 9.573E+00 | | set |
| $\mathrm{MONO}_{\mathrm{B0}}$ | blood monocytes (normalized) | 1.000E+00 | | set (normalized) |
| $\mathrm{L}_{\mathrm{B0}}$ | blood LDL-C (normalized) | 1.000E+00 | | set (normalized) |
| $\mathrm{MONO}_{\mathrm{Bnor}}$ | normal value of blood monocytes [$10^3$/ml] | 1.904E+02 | | set (from data) |
| $\mathrm{L}_{\mathrm{Bnor}}$ | normal value of blood LDL-C [mg/dl] | 1.132E+02 | | set (from data) |

## Functions $d_{in}$ for the various interventions.

In this chapter, we provide the jump functions $d_{\mathrm{in}}$ for scenarios 1–13. First column corresponds to the number of scenario from Table 2 with the intervention in column 1. The third column contains the time points at which the functions jump, and the fourth the associated function value in terms of $d_{\mathrm{HFD}}$, $d_{\mathrm{PA}}$, $d_{\mathrm{Immod}}$, and $d_{\mathrm{ABIO}}$.

| scenario | intervention | | week | parameter | |
| --- | --- | --- | --- | --- | --- |
| 1 | Control | $d_{\mathrm{in}}$=[ | 0 | 1 | |
| | | | 52 | 1 | ] |
| 2 | Control | $d_{\mathrm{in}}$=[ | 0 | 1 | |
| | | | 28 | 1 | ] |

| scenario | intervention | | week | parameter | |
| --- | --- | --- | --- | --- | --- |
| 3 | HFD | $d_{\mathrm{in}}$=[ | 0 | 1 | |
| | | | 8 | 1 | |
| | | | 8 | $d_{\mathrm{HFD}}$ | |
| | | | 52 | $d_{\mathrm{HFD}}$ | ] |
| 4 | HFD | $d_{\mathrm{in}}$=[ | 0 | 1 | |
| | | | 16 | 1 | |
| | | | 16 | $d_{\mathrm{HFD}}$ | |
| | | | 28 | $d_{\mathrm{HFD}}$ | ] |
| 5 | HFD | $d_{\mathrm{in}}$=[ | 0 | 1 | |
| | | | 8 | 1 | |
| | | | 8 | $d_{\mathrm{HFD}}$ | |
| | | | 16 | $d_{\mathrm{HFD}}$ | ] |

| scenario | intervention | | week | parameter | |
| --- | --- | --- | --- | --- | --- |
| 6 | HFD+ABIO | $d_{\mathrm{in}}$=[ | 0 | 1 | |
| | | | 12 | 1 | |
| | | | 12 | $d_{\mathrm{ABIO}}$ | |
| | | | 16 | $d_{\mathrm{ABIO}}$ | |
| | | | 16 | $d_{\mathrm{HFD}} \cdot d_{\mathrm{ABIO}}$ | |
| | | | 22 | $d_{\mathrm{HFD}} \cdot d_{\mathrm{ABIO}}$ | ] |
| 7 | HFD+PA | $d_{\mathrm{in}}$=[ | 0 | 1 | |
| | | | 16 | 1 | |
| | | | 16 | $d_{\mathrm{HFD}}$ | |
| | | | 18 | $d_{\mathrm{HFD}}$ | |
| | | | 18 | $d_{\mathrm{HFD}} \cdot d_{\mathrm{PA}}$ | |
| | | | 22 | $d_{\mathrm{HFD}} \cdot d_{\mathrm{PA}}$ | ] |
| 8 | ABIO+HFD+PA | $d_{\mathrm{in}}$=[ | 0 | 1 | |
| | | | 12 | 1 | |
| | | | 12 | $d_{\mathrm{ABIO}}$ | |
| | | | 16 | $d_{\mathrm{ABIO}}$ | |
| | | | 16 | $d_{\mathrm{ABIO}} \cdot d_{\mathrm{HFD}}$ | |
| | | | 18 | $d_{\mathrm{ABIO}} \cdot d_{\mathrm{HFD}}$ | |
| | | | 18 | $d_{\mathrm{ABIO}} \cdot d_{\mathrm{HFD}} \cdot d_{\mathrm{PA}}$ | |
| | | | 22 | $d_{\mathrm{ABIO}} \cdot d_{\mathrm{HFD}} \cdot d_{\mathrm{PA}}$ | ] |

| scenario | intervention | | week | parameter | |
| --- | --- | --- | --- | --- | --- |
| 9 | HFD+PA+Immod | $d_{\mathrm{in}}$=[ | 0 | 1 | |
| | | | 16 | 1 | |
| | | | 16 | $d_{\mathrm{HFD}}$ | |
| | | | 18 | $d_{\mathrm{HFD}}$ | |
| | | | 18 | $d_{\mathrm{HFD}} \cdot d_{\mathrm{PA}} \cdot d_{\mathrm{Immod}}$ | |
| | | | 22 | $d_{\mathrm{HFD}} \cdot d_{\mathrm{PA}} \cdot d_{\mathrm{Immod}}$ | ] |
| 10 | HFD+Immod | $d_{\mathrm{in}}$=[ | 0 | 1 | |
| | | | 16 | 1 | |
| | | | 16 | $d_{\mathrm{HFD}}$ | |
| | | | 18 | $d_{\mathrm{HFD}}$ | |
| | | | 18 | $d_{\mathrm{HFD}} \cdot d_{\mathrm{Immod}}$ | |
| | | | 22 | $d_{\mathrm{HFD}} \cdot d_{\mathrm{Immod}}$ | ] |

| scenario | intervention | | week | parameter | |
|---|---|---|---|---|---|
| 11 | Contr+ABIO | $d_{\text{in}}$=[ | 0 | 1 | |
| | | | 12 | 1 | |
| | | | 12 | $d_{\text{ABIO}}$ | |
| | | | 22 | $d_{\text{ABIO}}$ | ] |
| 12 | Contr+PA | $d_{\text{in}}$=[ | 0 | 1 | |
| | | | 18 | 1 | |
| | | | 18 | $d_{\text{PA}}$ | |
| | | | 22 | $d_{\text{PA}}$ | ] |
| 13 | Contr+PA+ABIO | $d_{\text{in}}$=[ | 0 | 1 | |
| | | | 12 | 1 | |
| | | | 12 | $d_{\text{ABIO}}$ | |
| | | | 18 | $d_{\text{ABIO}}$ | |
| | | | 18 | $d_{\text{ABIO}} \cdot d_{\text{PA}}$ | |
| | | | 22 | $d_{\text{ABIO}} \cdot d_{\text{PA}}$ | ] |

# Model analysis

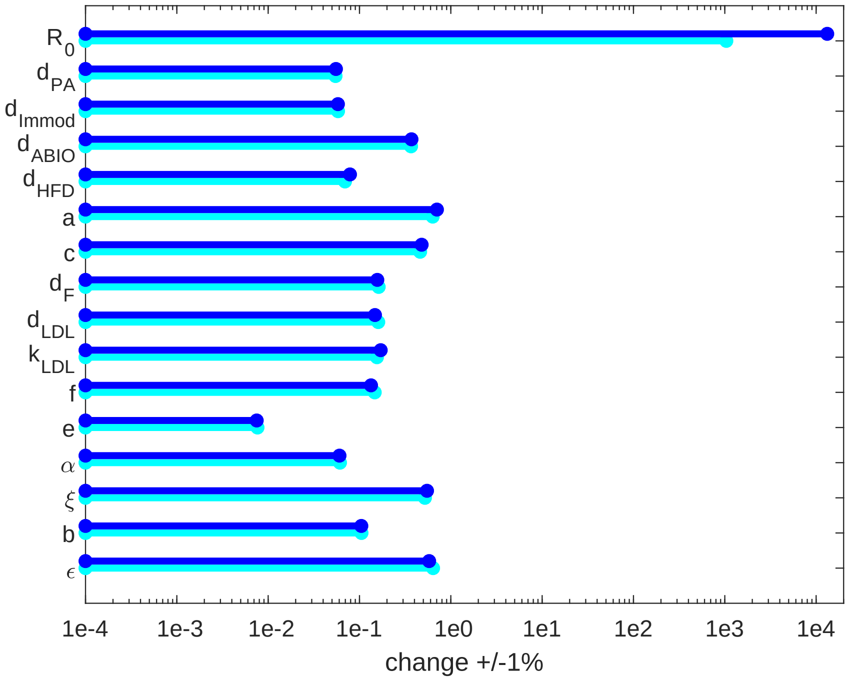


**Fig S1. Parameter sensitivity.** The horizontal bars show the deterioration of the fitness function (%) when the respective parameter value is increased (blue) or decreased (cyan) by 1%.

**Table S3. Steady state values after interventions. Resulting values of Lesion, IM, L, MONO, F, $MONO_B$, $L_B$ and R after a simulated period of two years using the intervention function $d_{in}$ for the five interventions Control, High-fat diet (HFD), IL-10 blocking medication (Immod), antibiotic treatment (Abio) and propionic acid (PA).**

| Group | Control | HFD | Immod | Abio | PA |
|---|---|---|---|---|---|
| Lesion | 17.225 | 49.158 | 61.093 | 65.116 | 5.875 |
| IM | 97.662 | 72.077 | 57.858 | 51.579 | 110.794 |
| L | 0.558 | 0.896 | 1.154 | 1.307 | 0.398 |
| MONO | 8.983 | 10.652 | 11.012 | 11.117 | 7.269 |
| F | 178.221 | 212.815 | 219.985 | 222.089 | 142.739 |
| $d_{in}$ | 1.000 | 1.326 | 1.626 | 1.810 | 0.884 |
| $MONO_B$ | 190.425 | 190.425 | 190.425 | 190.425 | 190.425 |
| $L_B$ | 113.187 | 150.128 | 184.037 | 204.884 | 100.109 |
| $R$ | 0.910 | 0.713 | 0.624 | 0.591 | 0.970 |

## Plaque development in mice

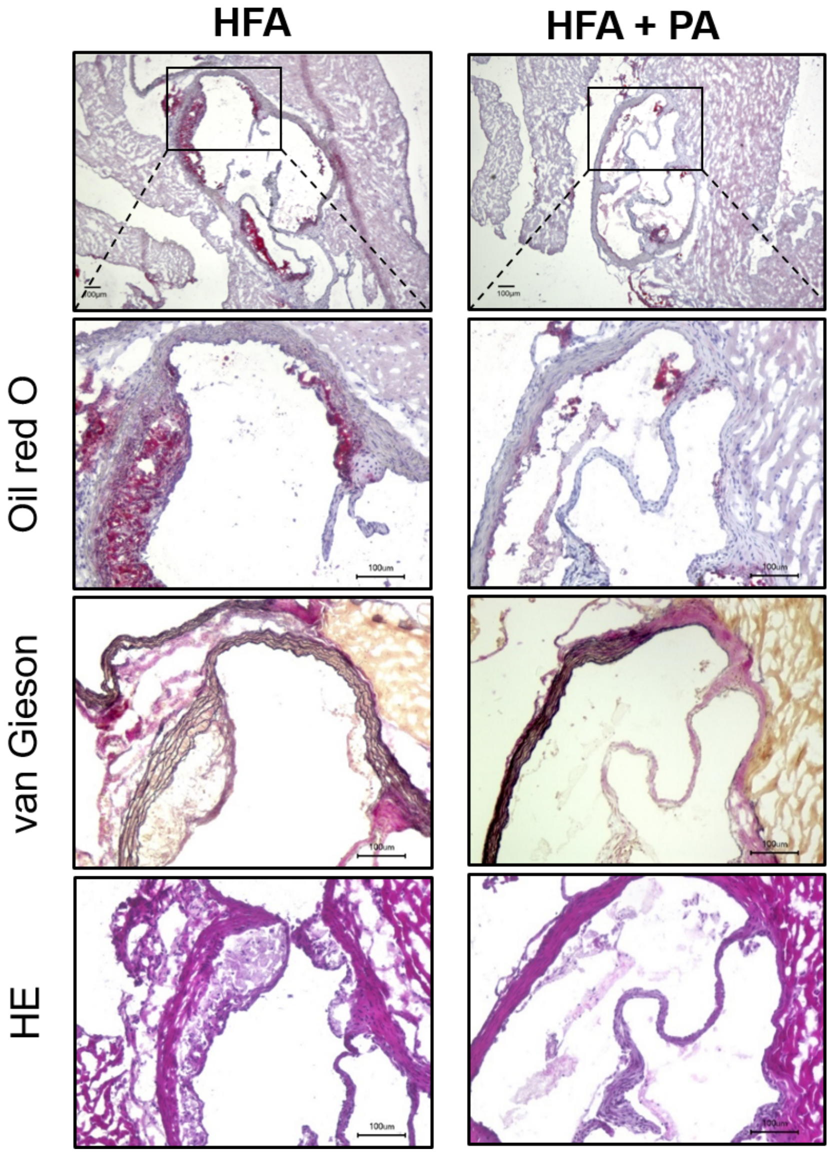


**Fig S2. Plaque characteristics.** We show representative sections of atherosclerotic plaques in APOE -/- mice fed with HFD without additional treatment (left column) and for HFD plus PA (right column). Plaque morphology was characterized by oil red o, van gieson and hematoxylin and eosin staining demonstrating predominantly inward growth of atherosclerotic plaques containing high levels of lipids.

# References

1. Bulelzai MAK, Dubbeldam JLA. Long time evolution of atherosclerotic plaques. Journal of Theoretical Biology. 2012;297:1–10.

2. Islam H, Johnston P. A mathematical model for atherosclerotic plaque formation and arterial wall remodelling. ANZIAM J. 2015;57:320. doi:10.21914/anziamj.v57i0.10386.
